# A time for action: antimicrobial resistance needs global response

**DOI:** 10.2471/BLT.16.181743

**Published:** 2016-08-01

**Authors:** Tim Jinks, Nancy Lee, Mike Sharland, John Rex, Nicholas Gertler, Matt Diver, Ian Jones, Kiron Jones, Sophie Mathewson, Francesca Chiara, Jeremy Farrar

**Affiliations:** a Wellcome Trust, Gibbs Building, 215 Euston Road, London NW1 2BE, England.; b Paediatric Infectious Diseases Unit, University of London, London, England.; c Astra Zeneca, Boston, United States of America (USA).; d Galen/Atlantica, Boston, USA.; e Jinja Publishing Ltd, London, England.

Given the magnitude and severity of the threat of antimicrobial resistance, it is a sign of progress that Member States of the World Health Organization (WHO) are now developing national action plans in response to WHO’s *Global action plan on antimicrobial resistance.*^1^ To accelerate these efforts, in April 2016 the Wellcome Trust held an interdisciplinary international summit, bringing together policy-makers and scientists from more than 30 countries to review and debate a set of 25 policy options.

The summit’s discussions reflected the multidimensional challenge posed by antimicrobial resistance. There are social, economic and environmental dimensions that encompass food production systems as well as human and animal health.^2^ Public attitudes and behaviours have a major impact on antibiotic use in health care.^3^ In many countries, agricultural use of antibiotics exceeds medical use.^4^ The solutions to antimicrobial resistance must be similarly broad in scope. The ‘One Health’ concept captures this scope, by recognizing the interdependence of human health, agriculture and animal health and the environment.

There are multiple tools and a growing knowledge base to enable national decision-makers to address antimicrobial resistance. Although evidence gaps have been cited as barriers to action,^5^ the summit concluded that knowledge gaps will always exist and that current evidence justifies immediate action. In particular, a range of policy interventions need to be implemented in three key areas.

First, antibiotic use in agriculture must be phased out without compromising the food system’s capacity to meet increasing global demand. The use of antibiotics for growth promotion and disease prevention should be phased out in favour of improved animal husbandry practices. Given the potential economic impact of such measures, particularly in low- and middle-income countries, insurance schemes could be developed to mitigate the risk to farmers of income loss through lower productivity during this transition. Research into alternative treatments and husbandry practices is required to support reduced antibiotic use in agriculture. Food production systems should also do more to limit consumer exposure to drug-resistant microbes.

Second, we need to develop a much better understanding of drug resistance levels and antibiotic use at the local level, in both human and animal medicine. Surveillance and monitoring are needed to provide a clear picture of local situations and to assess the impact of interventions. More comprehensive data are required on both antibiotic usage and resistance. Quantitative data will enable policy-makers to track the impact of interventions and set targets to motivate changes in behaviour, and will increase accountability. 

Third, public health systems need to optimize antibiotic use and reduce the disease burden. Consistent with the sustainable development goals, emphasis should be placed on improved sanitation and access to clean water, the promotion of good hand-hygiene practices and enhanced infection prevention and control in hospitals. By reducing infections and the need for antibiotics, these efforts would have an impact on antimicrobial resistance as well as delivering direct public health benefits. To promote these efforts, international development agencies need to include the prevention of antimicrobial resistance as a core aspect of their work. In addition, health worker education and professional development should have a stronger emphasis on antibiotic stewardship. Community-level education is necessary to ensure that all people understand what antibiotics can and cannot do and why minimizing use is in the interests of all. Over-the-counter access to antibiotics needs to be minimized. At the same time, many countries need to improve access to appropriate antibiotics. Measures are also needed to address the sale of antibiotics over the Internet. Financial incentives that link rewards to volumes of antibiotic sales also need to be eliminated.

Antimicrobial resistance affects every nation, but countries have varying needs and different capacities to address this challenge and face a multitude of competing health priorities. Nevertheless, every country can take actions that will directly benefit their own citizens while also helping to preserve our global antibiotic resources. Given that countries are at different stages in their development of response strategies, they should select policy interventions most appropriate to their circumstances, while building their capabilities over time. Sharing of information captured during implementation of policy interventions will build an evidence base to support local implementation.

The global community has sufficient tools and knowledge to manage antimicrobial resistance effectively – and thereby achieve a safer and healthier world for all. We have a shared responsibility to support all countries as they take the actions needed to safeguard the health of their citizens and combat the global threat posed by antimicrobial resistance.
